# Can Medical Humanities impart Empathy and Resilience skills to Medical Students?

**DOI:** 10.15694/mep.2020.000218.1

**Published:** 2020-10-06

**Authors:** Alan Schamroth, Hayley Berman, Neil Spencer

**Affiliations:** 1 University College London Medical School; 2 University of Hertfordshire

**Keywords:** Teaching Medical Humanities, Teaching Empathy and Resilience to medical students

## Abstract

This article was migrated. The article was marked as recommended.

The contribution of the Medical Humanities to a comprehensive medical education has been discussed elsewhere ( Schamroth, 2018), but what has been difficult to demonstrate is whether it has any measurable quantitative impact on improving student’s empathy or resilience. This small project was an attempt to further explore this question.

Medical students at University College London Medical School spend approximately one day a month during their first clinical year within a primary care setting in a programme called “Medicine in the Community” (MIC). The structure of the day involves students seeing patients under the supervision of primary care physicians. In this ethically approved research project (University College London, 2017) conducted over the academic year 2017-2018, a non-selected group of 24 students, received a compressed version of this MIC programme in the morning and in the afternoon were exposed to medical humanities. This included discussing poetry with a medical focus, creative writing based on the students own clinical experiences, watching and listening to carefully selected opera scenes where a health-related issue was illustrated and finally an experiential group based psychotherapy process using body mapping which facilitated the exploration of the interrelationship between mind and body.

A second group of 18 medical students who received the conventional MIC experience acted as the control. Both groups were given empathy and resilience questionnaires at the beginning and end of the academic year. The results showed that the students who experienced the afternoon humanities programme scored significantly higher in 3 of the 20 empathy questions than the control group and better in the resilience questionnaire, although the latter did not reach statistical significance.

## Introduction

In May 2017, following a number of high profile cases, the UK General Medical Council (GMC) in close partnership with the Academy of Medical Royal Colleges, responded by embedding common generic outcomes across all speciality post graduate medical curriculums. The result was the ‘Generic Professional Capability Framework’. This conceptual model of 9 domains included a fundamental core of professional values, behaviours and practice. Amongst these values was a duty to show respect, compassion and empathy for patients, carers and guardians and an ability to demonstrate emotional resilience ( GMC, 2017).

The GMC’s need to respond to shortcomings in postgraduate educational and training requirements, resonated with the first author who was concerned that although medical undergraduates were becoming increasingly knowledgeable they were in other ways ill equipped to face the messy complex emotional realities of illness, disability and death. He’d observed that students who often went into medical school straight out of secondary school, seemed to lacked the confidence, experiences of life and empathy to connect with patients which left patients frustrated and isolated. Others have noted that medical training had let many students down in failing to acknowledge their distress after witnessing traumatic and upsetting events in the teaching hospitals ( Schapiro, 2009) and that students were not given guidance on resilience skills to help them cope with a life in medicine to prevent distress, disillusionment and burnout ( Peters, 2018).

In response to these concerns, the authors devised a training programme using the medical humanities of art, opera, poetry and creative writing, to heighten the students’ sensitivity to how the patient might interpret their illness. The intension was to encourage the students to explore the bio-psychosocial impact of the illness on the patient and to use their own personal experience and/or imagination to cognitively identify with the patients and develop greater empathy. Running through the vertical programme of art, poetry, writing and opera, was a horizontal focus on groupwork and peer learning, playful creativity and learning from experience as a means of teaching resilience.

## Methods

Neither the student group of 24 who took part in the afternoon humanities training nor the 18 students in the control group, were selected. Students in both groups were routinely allocated and although not strictly randomised, there is no reason to suppose they differ in any way from the large 360 cohort of first clinical year medical students at University College London Medical School.

The 24 students in the humanities training experienced a less formal, but generally longer day to combine both traditional MIC activities and the humanities programme. Each component of the humanities programme was chosen to encourage empathy, teamwork, peer learning, creativity, curiosity, learning from success and failure and the acceptance of uncertainty.

The Poetry readings were selected from anthologies of health related poems (often written by patients) which described the subjective experience of acute illness, living with chronic disease (diabetes, depression, multiple sclerosis, rheumatoid arthritis) or facing death. The students were encouraged to imagine they were in the patients position and reflect on how they and their carers might have felt. The discussions allowed students to talk about illnesses in their own families or on the hospital wards and how the emotional impact affected them. This process draws on the key proponents of the value of group work of mirroring, resonance and reparation ( Foulkes, 1984).

The Creative Writing session followed, with students asked to use all five senses to actively record a single event on the hospital ward from the perspective of everyone present- the patient, carer, nurse, doctor, relative, porter or cleaner. This exercise required the students to imagine what all the participants in the clinical scenario were feeling, hearing, seeing, smelling and tasting. The model of practice within this framework is echoed by the methodology within the multidisciplinary Schwartz Centre Rounds where complex emotional and social care issues are presented from multiple perspectives. This model was piloted by the King’s Fund from 2009-13 and continued by The Point of Care Foundation. The results of these studies demonstrate increase in empathy, compassion and understanding, as well as preventing burn out in staff ( Goodrich, 2011).

Opera was the third component of the humanities programme and was the most challenging for the students because of its unfamiliarity. Here the students watched 20minute extracts from carefully selected operas in which a medical theme was explored and then the students were encouraged to discuss what emotions and feelings the opera aroused. The medium of opera was chosen because it offered multiple sensory stimuli- drama, music, voice, song, acting, passion & emotion. Themes covered included Violetta’s death from TB in Verdi’s La Traviata & Sour Angelica suicide in Puccini’s opera of the same name. The students were also asked to explore what the composer and librettist were trying to convey and whether they were successful. The opera’s chosen exposed students to the historic texture of patient and doctor dynamics, boundaries and responses. The viewing and listening offered an evocative space, expanding student’s potential to associate, learn and reflect.

Finally, the experiential art therapy sessions combined basic psychodynamic theory and counselling skills with art making. This involved student’s working and playing with multiple art media (paint, cloth, photos, print, cut out images & personal material) to develop ‘body-maps’. Here the students trace a life size outline of themselves which they then engaged with over the monthly sessions. The space within the outline represented the student’s image of themselves, their identity, aspirations and desires. The space beyond the outline represented the outside world with both its opportunities and constraints. In the same way that medical students are trained to take a history of patients, we asked our students to similarly take their own bio-psychosocial histories and explore their personal worries and concerns, their past histories (social, family, cultural, personal, medical) and only if it felt safe to then represent this is a pictorial, symbolic and creative form. Importantly the group thought about their body maps collectively, exploring symbolic meaning and this free associating opened up their curiosity and meaning making.

Both the humanities group of 24 students and the control group of 18 students were given empathy and resilience questionnaire tests at the beginning and end of the academic year. The empathy questionnaire which was completed anonymously was the Jefferson Scale of Empathy Medical Student version (JSE S-version) which is a 20 item questionnaire with a 7 point Likert scale scoring system meaning each question is scored from 1 (strongly disagree) to 7 (strongly agree) ( Hojat *et al.*, 2001). Permission to use the JSE questionnaire was requested and granted. This questionnaire was developed in response to a need for a psychometrically sound instrument and its validity and reliability is discussed in Chapter 7 of Dr M. Hojat’s book Empathy in Health Professions Education and Patient Care ( Hojat, 2016).

The 50 Item Resilience Questionnaire was taken from the Open Psychometric Test Resource which is a collaborative project from universities and their research students across the UK including Warwick, Southampton and Durham Universities ( The Psychometric Project, 2013). While the questionnaire was completed on-line the students’ data was then recorded anonymously is accordance with the ethics approval.

Each question is scored from 1 (very inaccurate) to 5 (very accurate). The 50 questions covered the 5 characteristics of adaptability, self-control, self-sufficiency, optimism and persistence. Adaptability refers to the ability to accept change and criticism and respond positively. Self-control is the likelihood of one’s emotions or desires to affect judgement, behaviour and ability to focus. Self-Sufficiency is the ability to work confidently and capably without being dependent or overly reliant on others. Optimism is the ability to look upon difficulties positively and with hope. Finally, Persistence is the ability to work through and persevere with hard, disappointing or challenging tasks.

## Results/Analysis

The data collected were for two groups (the 18 student control group and the 24 student humanities group) ‘before’ and ‘after’ the intervention (the humanities programme). In an ideal situation it should have been possible to link the ‘before’ and ‘after’ responses made by the same individual and thus calculate any change that has occurred at the individual level. However, since this data was collected anonymously, we do not have these identifiers and for each of the control and humanities groups have two non-independent sets of data: one for ‘before’ and one for ‘after’ the intervention.

The lack of independence here means that the analysis is more conservative than would be the case if we had information that allowed us to take account of the dependencies. In other words, any significant results discussed below would have p-values that were even smaller, had we been able to take account of the lack of independence. Also, in the analysis, a two-tailed test was used meaning that prior to analysis we made no assumption about whether the control or humanities group might yield higher or lower responses.

As a result of not being able to link ‘before’ and ‘after’ responses for individuals, we looked at how the control and humanities groups responses had changed as a whole between the ‘before’ and ‘after’ data collections. In order to compare these changes, we began by first assessing whether or not the control and humanities groups were similar in their responses at the ‘before’ stage (at the start of the year).

Table 1 gives summary statistics of the ‘before’ data for each group in terms of median and inter-quartile range and also gives a p-value resulting from a hypothesis test that considers whether or not the distribution of the responses is the same for the two groups. The continuous scale is because a large proportion of the responses are at the largest category of 7. This means that any assumption that the responses are realisations of a continuous phenomenon cannot be supported.

**Table 1.** Summary statistics for the Control and Experimental groups for the Empathy responses Before the intervention, with p-values

	Median, Inter-Quartile Range for Control Group	Median, Inter-Quartile Range for Experimental Group	p-value from Kruskal-Wallis test
Q1	6 [6, 7]	6 [6, 7]	0.544
Q2	6 [6, 7]	6 [6, 7]	0.917
Q3	5 [4, 5]	4 [4, 5]	0.177
Q4	6 [6, 7]	6 [5, 7]	0.833
Q5	5 [4, 5]	4 [4, 5]	0.902
Q6	5 [5, 6]	5 [4, 6]	0.719
Q7	7 [6, 7]	7 [6, 7]	0.673
Q8	7 [6, 7]	6 [6, 7]	0.639
Q9	6 [5, 7]	6 [6, 7]	0.238
Q10	6 [6, 7]	6 [6, 7]	0.989
Q11	6 [6, 6]	6 [5, 6]	0.933
Q12	7 [6, 7]	7 [6, 7]	0.386
Q13	7 [6, 7]	6 [6, 7]	0.408
Q14	7 [6, 7]	7 [6, 7]	0.673
Q15	6 [6, 7]	6 [6, 7]	0.598
Q16	6 [6, 7]	6 [6, 7]	0.374
Q17	6 [5, 6]	5 [5, 6]	0.838
Q18	4 [3, 4]	4 [4, 4]	0.043
Q19	7 [7, 7]	7 [5, 7]	0.005
Q20	7 [7, 7]	7 [6, 7]	0.253

From the p-values in Table 1, we can see that almost all are above the notional 5% (0.05) level for significance. Indeed, only one of the p-values (for Q19) is notably below this level. The small p-value for Q18 was not examined further because it was close to 5% and the fact that we are undertaking many tests means that one should employ a stricter cut-off than would otherwise be the case. For Q19, Table 2 below showed that the control group has its responses almost all in the highest category whereas the humanities group has a number of cases in slightly lower categories. Apart from this, the control and humanities groups do not differ greatly in terms of their responses before the intervention.

**Table 2.** Distribution of responses for Q19 for Control and Experimental groups

	Control	Experimental
1	0	0
2	0	0
3	0	0
4	0	3
5	0	4
6	1	4
7	16	13

In Table 3 which illustrates the comparisons of the control and humanities group responses ‘after’ the humanities programme, a number of empathy questions p-values are well below the 5%. Since multiple tests were carried out means that p-values not far below 5% should be disregarded. Nevertheless, there were 3 questions which showed much smaller p-values indicating a statistical significance. These are Q1, Q9 and Q18.

**Table 3.** Summary statistics for the Control and Experimental groups for the Empathy responses After the intervention, with p-values

	Mean, Median [Inter-Quartile Range] for Control Group	Mean, Median [Inter-Quartile Range] for Experimental Group	p-value from Kruskal-Wallis test
Q1	5.59, 6 [5, 6]	6.39, 6 [6, 6]	0.002
Q2	6.12, 6 [5, 7]	6.48, 7 [6, 7]	0.178
Q3	4.18, 4 [3, 5]	5.26, 5 [5, 5]	0.016
Q4	6.00, 6 [5, 7]	6.04, 6 [6, 7]	0.817
Q5	4.53, 5 [4, 5]	4.83, 5 [4, 5]	0.612
Q6	4.53, 4 [3, 6]	5.30, 5 [5, 6]	0.121
Q7	6.35, 7 [6, 7]	6.65, 7 [6, 7]	0.148
Q8	5.71, 6 [5, 6]	6.30, 7 [6, 6]	0.033
Q9	5.71, 6 [5, 6]	6.43, 7 [6, 6]	0.008
Q10	5.88, 6 [5, 6]	6.04, 6 [5, 6]	0.532
Q11	6.00, 6 [5, 7]	6.13, 6 [6, 7]	0.581
Q12	6.53, 7 [6, 7]	6.48, 7 [6, 7]	0.753
Q13	6.35, 6 [6, 7]	6.57, 7 [6, 7]	0.377
Q14	6.65, 7 [6, 7]	6.83, 7 [7, 7]	0.202
Q15	5.65, 6 [5, 7]	6.17, 6 [6, 7]	0.138
Q16	6.18, 6 [6, 7]	6.30, 6 [6, 7]	0.622
Q17	5.12, 5 [5, 6]	5.74, 6 [5, 6]	0.075
Q18	3.29, 3 [2, 4]	4.52, 4 [4, 4]	0.008
Q19	6.18, 6 [6, 7]	6.22, 7 [6, 7]	0.626
Q20	6.24, 6 [6, 7]	6.52, 7 [6, 7]	0.277

Q1 - ‘Physicians understanding of their patients’ feelings and the feelings of their patients’ families does not influence medical or surgical treatment’.

Q9 - ‘Physicians should try and stand in their patient’s shoes when providing care to them’.

Q18- ‘Physicians should not allow themselves to be influenced by strong personal bonds between their patients and their family members’.

According to the JSE questionnaire analysis, 3 meaningful factors/subscales emerged. These are ‘compassionate care’ which includes items 1 and 18, ‘perspective taking’ which includes items 9, and the third is “standing in the patient’s shoes’. However, the authors find is difficult to generalise on what these subscales mean in terms of their humanities programme and its teaching.

With regards to the resilience analysis, as with the empathy analysis, the first stage was to compare the control and humanities groups ‘before’ the humanities programme. Here the resilience responses can be treated as continuous and thus it is appropriate to calculate means and 95% confidence intervals. Further, the distribution is approximately Normal and comparisons of the control and humanities groups can be carried out using t-tests. Table 4 summarises the results for the five aspects of resilience ‘before’ the intervention. All the p-values are above 5% indicating that the two groups do not differ significantly before the humanities programme.

**Table 4.** Summary statistics for the Control and Experimental groups for the Resilience responses ‘Before’ the intervention, with p-values

	Mean, 95% CI for Control Group	Mean, 95% CI for Experimental Group	p-value from t-test
Adaptability	37.73 (35.25, 40.21)	37.13 (35.30, 38.96)	0.682
Self-Control	31.47 (28.49, 34.44)	30.74 (28.21, 33.26)	0.696
Self-Sufficiency	31.13 (28.41, 33.86)	31.35 (28.79, 33.90)	0.904
Optimism	40.13 (38.24, 42.03)	39.43 (37.67, 41.20)	0.573
Persistence	35.00 (33.43, 36.57)	34.91 (32.57, 37.26)	0.949

In Table 5, which summarises the two groups Resilience responses after the humanities programme, the p-values for all 5 measures is larger than 5% and thus we have insufficient evidence to claim a difference between the control and humanities groups. However, we note that for all measures, the means for the humanities groups were larger than for the control group (a situation that was only true for Self-Sufficiency before the humanities programme). For adaptability, the p-value is 0.067 and the mean for the humanities group is noticeably larger than for the control group, It is thus possible that the lack of statistical significance observed was due to there being insufficient data available to detect the difference between the two groups. If larger studies are undertaken, they may be able to resolve this issue.

**Table 5.** Summary statistics for the Control and Experimental groups for the Resilience responses ‘After’ the intervention, with p-values

	Mean, 95% CI for Control Group	Mean, 95% CI for Experimental Group	p-value from t-test
Adaptability	36.27 (33.62, 38.92)	39.04 (37.49, 40.60)	0.067
Self-Control	31.87 (28.60, 35.13)	33.52 (31.63, 35.42)	0.360
Self-Sufficiency	31.07 (28.52, 33.61)	32.70 (30.41, 34.98)	0.322
Optimism	39.33 (37.21, 41.45)	40.43 (38.53, 42.34)	0.420
Persistence	35.53 (33.63, 37.44)	35.96 (34.06, 37.86)	0.742

## Discussion

The terms empathy and resilience are both difficult to define, but there is a growing view that both are important attributes in clinicians with its obvious implications for medical education ( GMC, 2017). Since both terms are contested in definition and significance ( Smajdor, Stockl and Salter, 2011), it is important to consider our understanding of them. We have viewed empathy as the ability to stand in the patient’s shoes and ‘understand’ cognitively their thoughts, feelings and emotions through communication with an intension to help ( Hojat, 2016). This differs from sympathy where the clinician ‘shares’ the patient’s feelings of sorrow or feels pity for the patient.

With regards to resilience, we use the three element conceptualisation which views resilience as 1) an ongoing process of adaptation and flexible responses to environmental challenges, 2) involving both individual effort (self-efficacy) and mutually supportive relationships (peer, professional and personal) and 3) developing the capacity for creativity (in the good times) to be able to thrive when faced with challenges ( Beltman, Mansfield and Price, 2011). This contains similarities to the General Medical Council’s definition of emotional resilience as ‘the ability to adapt and be resourceful, mindful and effective in complex, uncertain or stressful situations or crisis’ ( GMC, 2017).

The results show a statistically significant increase in 3 out of 20 questions in the Jefferson Scale of Empathy Student version. The Resilience 50 Item Questionnaire results showed a mean increase across all 5 characteristics of the humanities group compared to the control group, although it did not reach statistical significance. To explain these findings we looked at the context, content and process of the humanities programme and how all may have impacted on the students learning.

The context within which this humanities programme occurred undoubtedly played an important and influential role in creating the mood & atmosphere of the experience. The humanities groups of 24 students spent the day in a less formal setting which included a communal lunch whereas the control group spent the day in a more conventional setting. Although the formal humanities training began in the afternoon, the students experienced a compressed conventional morning session to accommodate the traditional elements of the ‘Medicine in the Community’ course so as not to sacrifice any traditional learning. Since the conventional morning clinical experience incorporated a practical focus on exploring the patients psychosocial and emotional response to their illness, it could be argued that the whole day was focussed on developing and delivering an empathic model. While it is true that all medical students are required to inquire about psycho-social aspects of illness, the students’ anecdotal feedback of experience in hospitals suggests this requirement may not be given the importance it deserves.

The process of developing this humanities programme began with a general observation of the difficulties faced by students when dealing with the psycho-social elements of Doctor-Patients consultations and their reluctance to expose any personal emotional vulnerability by adopting a professional veneer at all times. The programme design was to enhance students’ confidence and capacity for accepting their own emotions so they would not feel inhibited from engaging empathically with patients. The authors brought to this programme an enthusiasm, belief and commitment that teaching resilience and empathy was not only necessary to encourage students to be curious about the patients’ inner world, but possible. This aim was imbedded in the teaching of the humanities by ensuring that this strand ran right across the poetry, creative writing, opera and art. This horizontal thread highlighted an empathic understanding of the patient’s cognition, behaviour and emotions and encouraged resilience in the students to problem solve, develop self-care practices, communicate and work with peers to develop purposefulness, optimism and persistence.

The content of the day was focussed on developing both empathy and resilience with the medical humanities as the medium. Each of the different modalities was taught by a specialist. The poetry was taught by a trained reading educator, the creative writing by a poetry and language teacher, the opera by a lay opera educator and the practical art by an academic art psychotherapist. In addition to the formal humanities programme the students were set clinical homework between the monthly sessions which required collaboration and co-operation. This collaborative effort required the students to value each other’s contribution, communicate with and support each other during the time between sessions. The authors thought it was important to demonstrate how peer support allowed the groups to learn from and appreciate each other and act as a model for future professional collaboration. This close working relationship based on trust and interdependency and the non-hierarchical nature of the collaboration, may have strongly influenced the confidence with which the students’ shared thoughts and emotions and increased their capacity for thriving.

## Conclusion

While it is generally believed that empathy and resilience are necessary and desirable skills for an effective, long and successful medical career ( GMC, 2017), it cannot and must not be assumed that these skills are automatically acquired during medical student training. Since there is a growing acceptance that these skills can be learnt, an important question is how are they taught? In this paper we used the medium of the medical humanities to teach empathy and resilience skills and demonstrated a statistical significance in 3 out of 20 questions of the Jefferson Empathy Questionnaire and although the resilience questionnaire didn’t reach statistical significance, each one of 5 resilience characteristics received an averaged higher score in the humanities group. If this admittedly small study had been of a higher power (a larger study) then statistical significance may have been demonstrated here too. The challenge ahead is how to scale up this teacher intensive programme for the whole student cohort in an already overloaded curriculum and a resource constrained environment. The former point was achieved in this programme, by using the teaching time more efficiency and we believe more effectively. The issue of providing the financial resources to embed humanities teaching in the curriculum, requires a political will and a belief that the medical humanities offers value in applying a broader psychosocial and psychoanalytical understanding to the complexity of being a doctor. We believe this approach will deepen students understanding of the importance of the relationship between the patient and doctor and expose how their own internal struggles impact the patients as well as how patients physical and emotional distress impact students. Through the humanities the students were able to explore the psychosomatic presentation of illness that have links to emotion and historical residue that can be creatively and gently held in the consultation process. The students were able to be more mindful of the importance of listening to their patients from the outside in and inside out.

We feel that the humanities provide a helpful and useful toolkit for establishing boundaries while staying curious, engaged and open and of offering opportunities to both develop empathy and grow resilience.

## Take Home Messages

• Empathy and resilience skills can and should be taught to medical students.
• The Medical Humanities are a useful medium for delivering these skills.

## Notes On Contributors

**Dr Alan Schamroth** is a General Medical Practitioner in North London and at the time of this research project was an Associate Member of Department of Primary Care and Population Health, University College London Medical School. Dr Schamroth is a Primary Care Physician and teaches first year clinical medical students from University College London Medical School.

**Dr Hayley Berman** is a Senior Lecturer in Art Therapy and at the time of this research project was Programme Leader MA Art Therapy, School of Creative Arts, University of Hertfordshire. Dr Berman is senior lecturer on the MA Art Therapy programme and supervisor and examiner of PhD students for Public Health, Psychosocial Studies and Visual Arts at University of Hertfordshire and Visiting Professor at the University of Johannesburg.

**Professor Neil Spencer** is Professor in Applied Statistics, Hertfordshire Business School, University of Hertfordshire and Director of its Statistical Services and Consultancy Unit.
