# Supplementary material for: Can Medical Humanities impart Empathy and Resilience skills to Medical Students?
Source: MedEdPublish (2016). 2020 Oct 6;9:218. [Version 1] doi: 10.15694/mep.2020.000218.1 (PMC10699361; doi:10.15694/mep.2020.000218.1)
Supplement: Peer review history

# Reviewer report: Reviewer response for version 1

**DOI:** 10.21956/mep.20128.r31062

**Published:** 2020-10-22

**Authors:** Ryan Kane

**Affiliations:** 1 Oregon Health & Science University

This review has been migrated. The reviewer awarded 4 stars out of 5 Schamroth et al. created an impressive medical humanities program to enhance empathy and resilience among early medical trainees. Though this study had a relatively small sample size, employed a singular follow up to assess efficacy, and did not reach statistical significance in most of the analyzed fields, it benefitted from notable curricular time allocation for the medical humanities, had a robust curriculum with diverse activities, utilized pre-validated questionnaires, and employed a pre-/post-analysis strategy to statistically assess program efficacy. It would have been interesting to see if these trainees had a difference in qualitative metrics and/or if the study generated divergent empathy/resilience scores as they continued through training. I look forward to future studies from this group.

Reviewer Expertise:

NA

No decision status is available

# Reviewer report: Reviewer response for version 1

**DOI:** 10.21956/mep.20128.r31063

**Published:** 2020-10-09

**Authors:** Subha Ramani

**Affiliations:** 1 Harvard Medical School, Brigham and Women's Hospital

This review has been migrated. The reviewer awarded 4 stars out of 5 In an era where fostering resilience among medical trainees and practising clinicians is taking centre-stage, this paper adds some interesting insights. The authors are correct in querying whether medical humanities can impact students’ empathy or resilience. They use interesting range of activities such as creative writing, watching opera scenes and body mapping. I was not aware of the last modality and read with interest.The introduction was a very nice balance of personal reflections (concerns about capacity of learners to face emotional realities of healthcare), literature reports and organizational mandates (Generic professional capability framework).The authors describe their intervention very well under methods- the poetry readings, creative writing with the use of all five senses, opera watching and experiential art therapy sessions. Very interesting and multi-modality approach to humanities training and I learned new things that I would like to apply in my own teaching. I had never heard of body mapping before.The measurements along the empathy scale and comparisons between control and intervention groups are shown in the results section.A few comments: statistical significance may not always be the yardstick, meaningful change is intangible and authors can reflect on how to showcase this. This is a good pilot but I would emphasize that humanism and resilience can be quickly extinguished based on the hidden curriculum students observe in actual clinical practice. Institutions should consider longitudinal training and role-modeling by clinical supervisors which likely needs faculty development.I agree with most of the reflections stated by the authors, more work needs to be done in this area and with practicing clinicians who face the brunt of the stresses of clinical practice.All those interested in the area of empathy and resilience will find this article useful and applicable to their own initiatives.

Reviewer Expertise:

NA

No decision status is available

# Reviewer report: Reviewer response for version 1

**DOI:** 10.21956/mep.20128.r31064

**Published:** 2020-10-07

**Authors:** P Ravi Shankar

**Affiliations:** 1 American International Medical University

This review has been migrated. The reviewer awarded 4 stars out of 5 As an educator with a keen interest in the health humanities I read the article with interest. The authors developed a medical humanities program for medical students and had a control group of students also. They showed empathy scores increased among the test group and there was also an increase in resilience scores. Further details about the actual program will be of interest. Where was the sessions held and how were the logistics organized? I found the creative writing session using all five senses and the body maps to be especially interesting. I do agree with the authors about the challenges of scaling up the program and offering it to all students. Commitment of time and resources to the medical humanities remains a challenge. I assume these were undergraduate medical students. I would also be interested in knowing about the exact role of the Academy of Medical Royal Colleges. This is an interesting article and will add to the increasing body of evidence being created about the usefulness of the medical and health humanities.

Reviewer Expertise:

NA

No decision status is available
